# An Algorithm that Predicts the Viability and the Yield of Human Hepatocytes Isolated from Remnant Liver Pieces Obtained from Liver Resections

**DOI:** 10.1371/journal.pone.0107567

**Published:** 2014-10-14

**Authors:** Serene M. L. Lee, Celine Schelcher, Rüdiger P. Laubender, Natalja Fröse, Reinhard M. K. Thasler, Tobias S. Schiergens, Ulrich Mansmann, Wolfgang E. Thasler

**Affiliations:** 1 Department of General, Visceral, Transplantation, Vascular and Thoracic Surgery, Grosshadern Hospital, Ludwig Maximilians University, Munich, Germany; 2 Tissue Bank, Department of General, Visceral, Transplantation, Vascular and Thoracic Surgery, Grosshadern Hospital, Ludwig Maximilians University, Munich, Germany; 3 Institute for Medical Information Processing, Biometry and Epidemiology, Grosshadern Hospital, Ludwig Maximilians University, Munich, Germany; Bambino Gesu' Children Hospital, Italy

## Abstract

Isolated human primary hepatocytes are an essential *in vitro* model for basic and clinical research. For successful application as a model, isolated hepatocytes need to have a good viability and be available in sufficient yield. Therefore, this study aims to identify donor characteristics, intra-operative factors, tissue processing and cell isolation parameters that affect the viability and yield of human hepatocytes. Remnant liver pieces from tissue designated as surgical waste were collected from 1034 donors with informed consent. Human hepatocytes were isolated by a two-step collagenase perfusion technique with modifications and hepatocyte yield and viability were subsequently determined. The accompanying patient data was collected and entered into a database. Univariate analyses found that the viability and the yield of hepatocytes were affected by many of the variables examined. Multivariate analyses were then carried out to confirm the factors that have a significant relationship with the viability and the yield. It was found that the viability of hepatocytes was significantly decreased by the presence of fibrosis, liver fat and with increasing gamma-glutamyltranspeptidase activity and bilirubin content. Yield was significantly decreased by the presence of liver fat, septal fibrosis, with increasing aspartate aminotransferase activity, cold ischemia times and weight of perfused liver. However, yield was significantly increased by chemotherapy treatment. In conclusion, this study determined the variables that have a significant effect on the viability and the yield of isolated human hepatocytes. These variables have been used to generate an algorithm that can calculate projected viability and yield of isolated human hepatocytes. In this way, projected viability can be determined even before isolation of hepatocytes, so that donors that result in high viability and yield can be identified. Further, if the viability and yield of the isolated hepatocytes is lower than expected, this will highlight a methodological problem that can be addressed.

## Introduction

The liver carries out a diverse range of necessary functions, such as homeostasis, metabolism and detoxification. As much of the research on the liver is human-centric, whether for the elucidation of mechanisms, translational research or cell-based therapy, isolated human liver cells remain an important *in vitro* model for basic and translational research.

One of the main uses of a human *in vitro* hepatocyte model is for the validation of studies done using animal models due to species differences. Olson *et al.*, [1] showed that when 150 drugs that cause human toxicity are tested, the concordance between toxicity found in animal studies and that observed in clinical practice is 70%. Similarly, Brambilla and Martelli found that when 42 compounds from various chemical families were tested for their toxicity in rat or human hepatocytes, 28 had similar toxicities, 10 were more toxic for rats, 3 were moderately more toxic for human hepatocytes and 1 was lethal for rat hepatocytes at a concentration 30-fold lower than that equally toxic for human hepatocytes [2]. In addition, animal models could also have less genetic variation than humans; Brambilla and Martell [2] found that the inter-individual variability in hepatocyte responses to chemicals that cause cytotoxicity is greater for humans than for rats. This would make it harder to detect idiosyncratic drug-induced liver injury.

The usage of human hepatocytes comes with the additional advantage of following the 3R ethical framework [3] to replace the use of research animals when possible. This is as the liver tissue used in this study was obtained from human elective liver resections. After resection, the tissue was immediately brought to a pathologist, who would take what was required for histopathological evaluation. The rest of the tissue, which is not needed, was designated as surgical waste. If a patient had signed an informed consent [4], this discarded tissue could then be collected for hepatocyte isolation.

In order to successfully use human hepatocytes as an *in vitro* model or for cell-based therapy, hepatocytes must be obtained with good viability and hence quality. Further, as the sources of human hepatocytes are limited and the cost of the entire process from informed consent to successfully obtaining hepatocytes is high, it is also important to understand the factors that can result in a compromised yield. Thus far, the literature on factors that affect viability and yield is contradictory (Tables 1 and 2). Further, the previously done studies had a small sample size ranging from 10 to 149 donors. Therefore, this study aimed to determine, with a large number of donors and hepatocyte isolations carried out over 10 years, the donor characteristics, medical histories and operation, tissue processing and cell isolation parameters that affect the viability and the yield of isolated human hepatocytes.

**Table 1:** Summary of factors affecting the viability of isolated hepatocytes.

Variables	Decreased viability	No change in viability	Increased viability
Age	Increased age [12], [38]	Different ages [15], [39]	Decreased age* [16]
Gender		Male or female [12], [13], [39]	
Fibrosis	Fibrotic organs [39]		Organs that are not steatotic, fibrotic or cirrhotic [39]
Cirrhosis	Cirrhotic organs [39]		Organs that are not steatotic, fibrotic or cirrhotic [39]
Steatosis		Severely steatotic organs or organs that are not steatotic, fibrotic or cirrhotic [39]	Visually steatotic liver [12]
Disease	Malignant disease [15]	Colonic secondary, cholangiocarcinoma, carcinoid, hepatocellular tumour, unknown primary, hyatid cyst, lung secondary or multi-organ donors [12], primary biliary cirrhosis or primary sclerosing cholangitis [9]	Benign disease [15]
Chemotherapy		Treated or untreated [14]	
Serum enzymes	Increased pre-operative GGT levels [15]		
Operation type		Right hepatectomy, segmental resection, left hepatectomy, extended right, local excision or multi-organ donor [12]	
Warm ischemia	Increased time* [16]	Varying time [11], no pringle or varying Pringle times [12]	
Cold ischemia	>20 h [39]	Up to 4 h [12], varying times [38], [39]	<10 h [39]

*Statistics done with multiple regression analysis. All other variables were analysed using univariate analyses.

**Table 2:** Summary of factors affecting the yield of isolated hepatocytes.

Variables	Decreased yield	No change in yield	Increased yield
Age	>50 years old [15]	Different ages [10], [13], [39], [40]	
Gender		Male or female [12], [13], [39]	
Fibrosis	Fibrotic organs [39]		Organs that are not steatotic, fibrotic or cirrhotic [39]
Cirrhosis	Cirrhotic organs [39]		Organs that are not steatotic, fibrotic or cirrhotic [39]
Steatosis	Severely steatotic organs [39]	No steatosis, <10% steatosis or >10% steatosis [10], no steatosis or >10% steatosis [13]	Organs that are not steatotic, fibrotic or cirrhotic [39]
Disease	Malignant disease [15]	Benign hepatic diseases, metastases from colorectal cancer, hepatic primitive malignant tumours or metastases from non-colorectal cancer [10], colonic secondary, cholangiocarcinoma, carcinoid, hepatocellular tumour, unknown primary, hyatid cyst, lung secondary or multi-organ donors [12], benign hepatic disease, metastases from colorectal cancer, hepatic primitive malignant tumours or metastases from non-colorectal cancer [13], primary biliary cirrhosis or primary sclerosing cholangitis [9]	Benign disease [15]
Chemotherapy		Treated or untreated [10], [14]	
Serum enzymes	Increased pre-operative GGT levels [10]	Increased pre-operative ALT or AST levels [10]	
Operation type		Right hepatectomy, segmental resection, left hepatectomy, extended right, local excision or multi-organ donor [12]	
Warm ischemia	Intermittent clamping [13]	Varying time [11],no pringle or varying Pringle times [12], no clamping, continuous or intermittent clamping [13]	No clamping [13]
Cold ischemia		Up to 4 h [12], up to 5 h [13], varying times [39]	
Perfused liver	100–200 g liver pieces [12], increased liver weight [16]	Varying weights [10], [13]	

## Material and Methods

### Ethics Statement

The liver pieces used for hepatocyte isolation were collected from resected liver specimens designated as surgical waste after examination by a pathologist. In particular, the tissue used was dissected from the resection margin of tumours containing morphologically healthy tissue. All liver pieces were collected with their associated clinical data by the Tissue Bank under the Administration of the Human Tissue and Cell Research (HTCR) Foundation (http://www.htcr.de/english/contacts.html) [4]. The HTCR-process included written informed consent, was approved by the Ethics Committee of the Medical Faculty of Regensburg University Hospital (approval number 99/46) and Ethics Committee of the Medical Faculty of Ludwig Maximilians University (approval number 025-12) and complied with the Bavarian Data Protection Act.

### Patients

In total, hepatocytes were isolated from remnant liver pieces from 1034 patients from Regensburg University Hospital (December 1997 to December 2002 and July 2010 to December 2011) and Grosshadern Hospital located in Munich (January 2003 to December 2013).

Corresponding data on the donor characteristics, medical histories and operation, tissue processing and cell isolation parameters were collected and entered into a database. The variables of interest for this study are listed on Table 3.

**Table 3:** Variables considered for statistical analyses.

Variables	Categories	Abbreviation	Unit
**Donor characteristics**			
Age		-	-
Gender	Male or female	-	-
Body mass index		BMI	-
Fibrosis	Yes or no	-	-
Cirrhosis	Yes or no	-	-
Diabetes	Yes or no	-	-
Obesity	Yes or no	-	-
Hypertension	Yes or no	-	-
Hypercholesterolemia	Yes or no	-	-
Hyperuricemia	Yes or no	-	-
Smoking	Yes, no or ex-smoker	-	-
Cigarettes per day		-	-
Liver fat	Yes or no	-	-
Liver fat		-	%
Tumour type	Benign or malignant	-	-
Surgical indication	Hepatocarcinoma (HCC), metastasis, focal nodular hyperplasia (FNH), klatskin, adenoma, cholangiocarcinoma (CCC) or others	-	-
Chemotherapy	Treated or untreated	-	-
ASA physical status classification system	1, 2, 3 or 6	ASA	-
Ludwig score	No or minimal fibrotic changes, periportal fibrosis, septal fibrosis or cirrhosis	-	-
**Clinical chemistry results before operation**			
Alkaline phosphatase activity		AP	U/L
Aspartate aminotransferase activity		GOT	U/L
Gamma-glutamyltranspeptidase activity		GGT	U/L
Alanine aminotransferase activity		GPT	U/L
Cholinesterase activity		CHE	U/L
Bilirubin		-	mg/dL
Partial thromboplastin time		PTT	s
Quick value		-	%
**Operation parameters**			
Operation type	Hemihepatectomy right (HR), Hemihepatectomy left (HL), segment resection (SR), atypical resection (AR) extended hepatectomy (EH), liver transplantation (LT) or lobectomy (L)	-	-
Warm ischemia *in vivo*		-	min
Warm ischemia *ex vivo*		-	min
Weight of resected liver		-	g
**Tissue processing and cell isolation parameters**			
Cold ischemia		-	min
Weight of perfused liver		-	g

### Isolation of Human Hepatocytes

Primary human hepatocytes were isolated using a two-step collagenase perfusion technique [5], [6] with modifications [7]. In short, the larger blood vessels on a liver piece with one cut face were cannulated with irrigation cannulae with olive tips. The liver piece was then perfused first with 1 L of Solution 1, which contains 154 mM sodium chloride, 20 mM HEPES, 5.6 mM potassium chloride, 5 mM glucose and 25 mM sodium hydrogen carbonate. Next, it was perfused for 10 min with Solution 2 (152.5 mM sodium chloride, 19.8 mM HEPES, 5.5 mM potassium chloride, 5 mM glucose and 24.8 mM sodium hydrogen carbonate and 0.1 mM EGTA) followed by Solution 3 (152.5 mM sodium chloride, 19.8 mM HEPES, 5.5 mM potassium chloride, 5 mM glucose and 24.8 mM sodium hydrogen carbonate and 0.5 µM calcium chloride dihydrate) for 0.5 L. Finally, it was perfused with Solution 4 (120 mM sodium chloride, 10 mM HEPES, 0.9 mM calcium chloride dehydrate, 6.2 mM potassium chloride and 0.1% w/v albumin), which contains 0.1 to 0.15% w/v collagenase for 9 to 12 minutes or until the liver is sufficiently digested. The liver piece was then placed carefully in a crystallising dish for removal of the Glisson's capsule before gently shaking the cells loose. The cell suspension was then filtered through a 210 µm nylon mesh followed by a 70 µm nylon mesh before centrifuging at 72 g for 5 min at 4°C to pellet the hepatocytes. Hepatocytes were then washed 3 times before resuspending the cells in Cold Storage Solution (Hepacult GmbH, Germany).

A hemocytometer-based trypan blue dye exclusion assay was done to quantify the viability and total cells yielded by this isolation procedure.

### Statistical Analyses

The data were summarised by adequate measures of location and spread. For modelling the outcomes of “viability” and “yield”, linear regression modelling was used when the variables in Table 3 were considered.

To account for the possibility of non-linear relationships between the considered outcome and the continuous covariates (Table 3), fractional polynomials of first and second degree were applied. For this purpose, the multivariable fractional polynomials (MFP) algorithm [8] was used. This algorithm combines the selection of the functional forms of each continuous covariate using fractional polynomials with the selection of all continuous and non-continuous covariates via backward elimination. For the multiple regression models, only donors with a complete set of information for the variables of interest were used. Therefore, 218 donors were considered for viability and 128 donors were considered for yield. The selection level for potential predictors was set to 0.05.

In order to satisfy the assumption of normality, viabilities were transformed by applying the logit and yields by applying the fourth root. Graphical procedures were used to assess the fit of the model. All tests were performed two-sided and a p-value lower than 0.05 was considered statistically significant. For all statistical analyses the software R (version 2.13.1) was used.

## Results

A total of 1034 hepatocyte isolations were done with an average viability of 78±10% and average yield of 13±11 million viable hepatocytes per gram liver with the values represented in means ± standard deviation.

### Univariate analyses to determine relationships of variables to viability and yield of hepatocytes

After linear regression analyses were carried out, variables with or without a significant relationship to the viability and the yield of hepatocytes were listed in Tables 4 and 5 respectively. In addition, figures were generated for the variables with significant relationships to the viability (Figures 1 to 4) and the yield (Figures 5 to 9) of hepatocytes.

**Figure 1:**
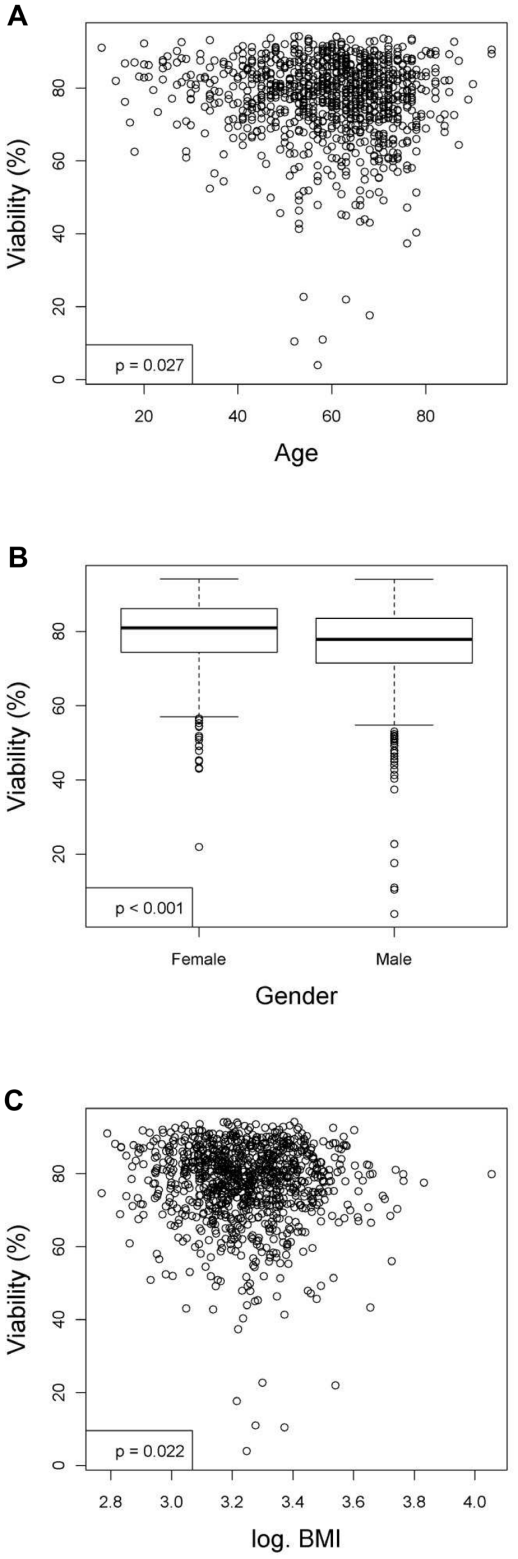
Donor characteristics that have significant relationships with the viability (%) of hepatocytes after linear regression analyses. Figures show relationships between viability and (**A**) age, (**B**) gender or (**C**) body mass index (BMI). Values were deemed significant when P<0.05.

**Figure 2:**
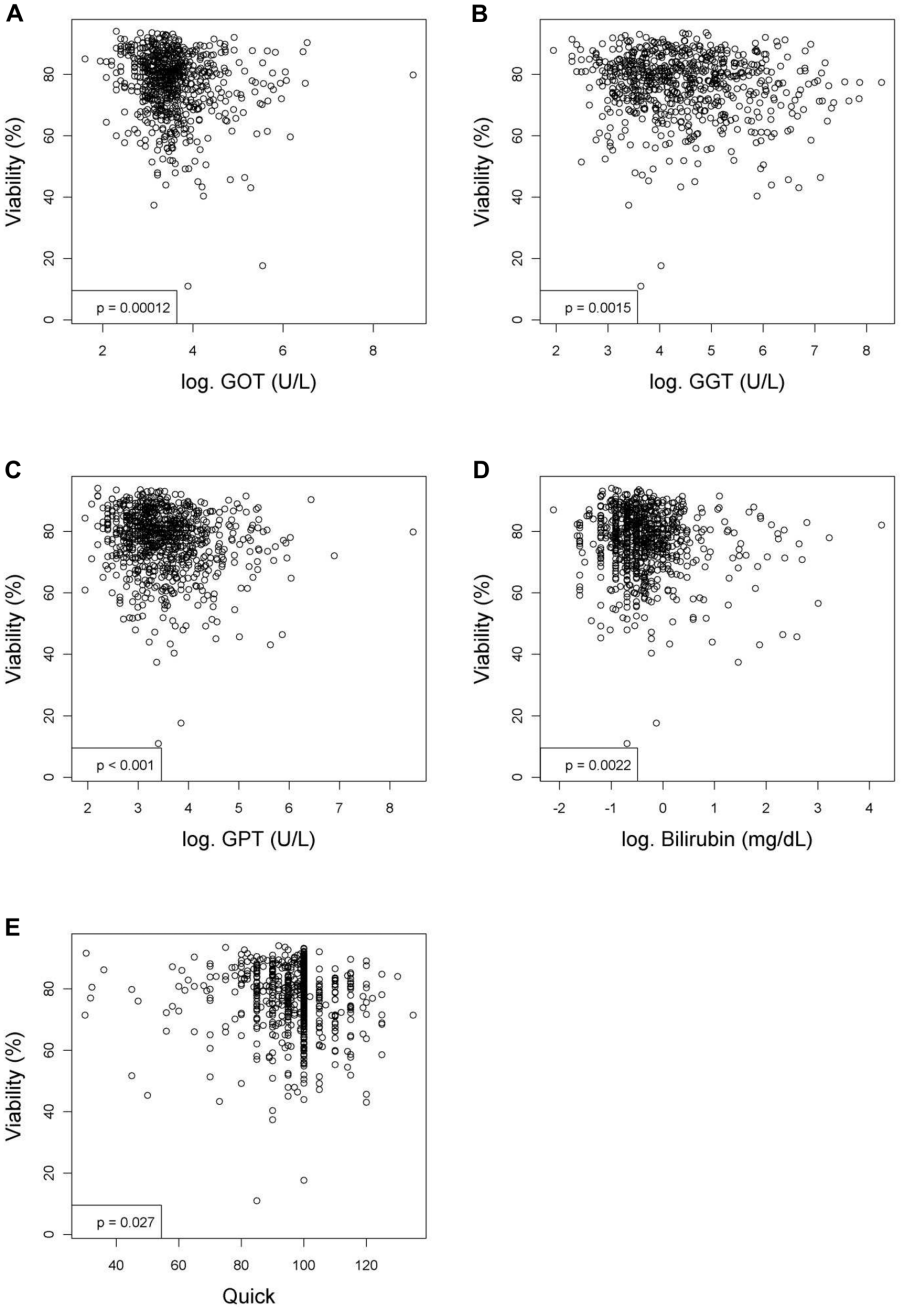
Variables measured in the blood or serum that have significant relationships with the viability (%) of hepatocytes after linear regression analyses. Figures show relationships between viability and (**A**) aspartate aminotransferase activity (GOT; U/L), (**B**) gamma-glutamyltranspeptidase activity (GGT; U/L), (**C**) alanine aminotransferase activity (GPT; U/L), (**D**) bilirubin (mg/dL) or (**E**) quick value (%). Values were deemed significant when P<0.05.

**Figure 3:**
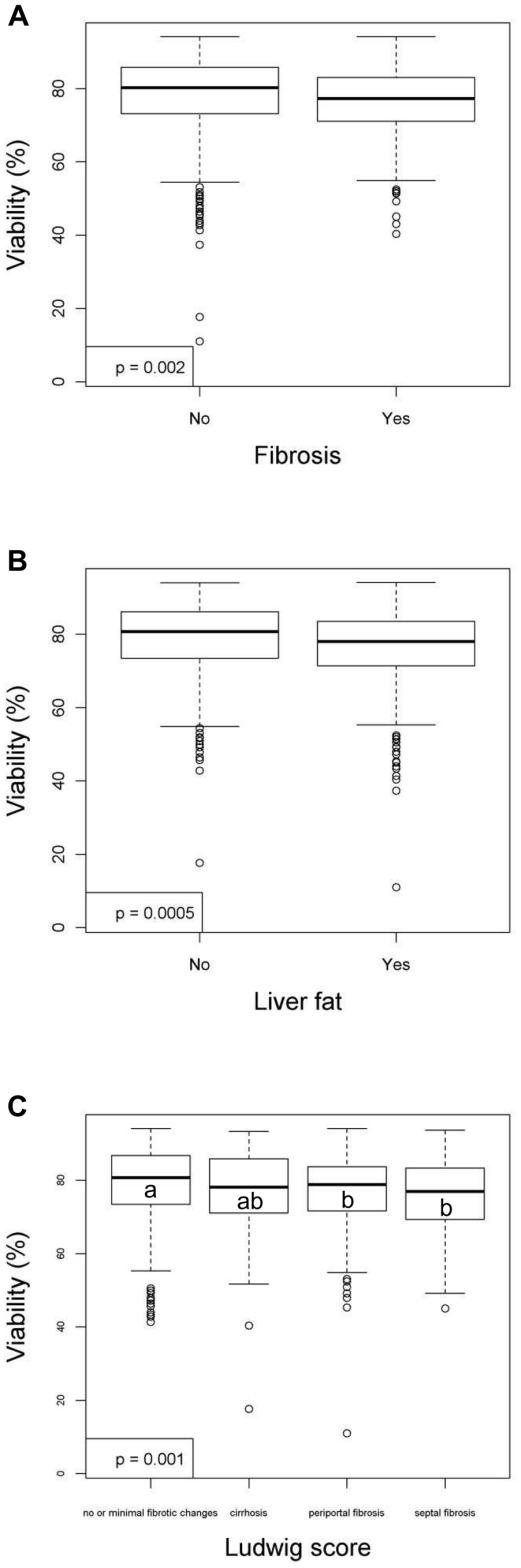
Liver variables that have significant relationships with the viability (%) of hepatocytes after linear regression analyses. Figures show relationships between viability and (**A**) fibrosis, (**B**) liver fat or (**C**) Ludwig score. Values were deemed significant when P<0.05. For the variable of Ludwig score, variables not sharing the same alphabet are significantly different, *P*<0.05.

**Figure 4:**
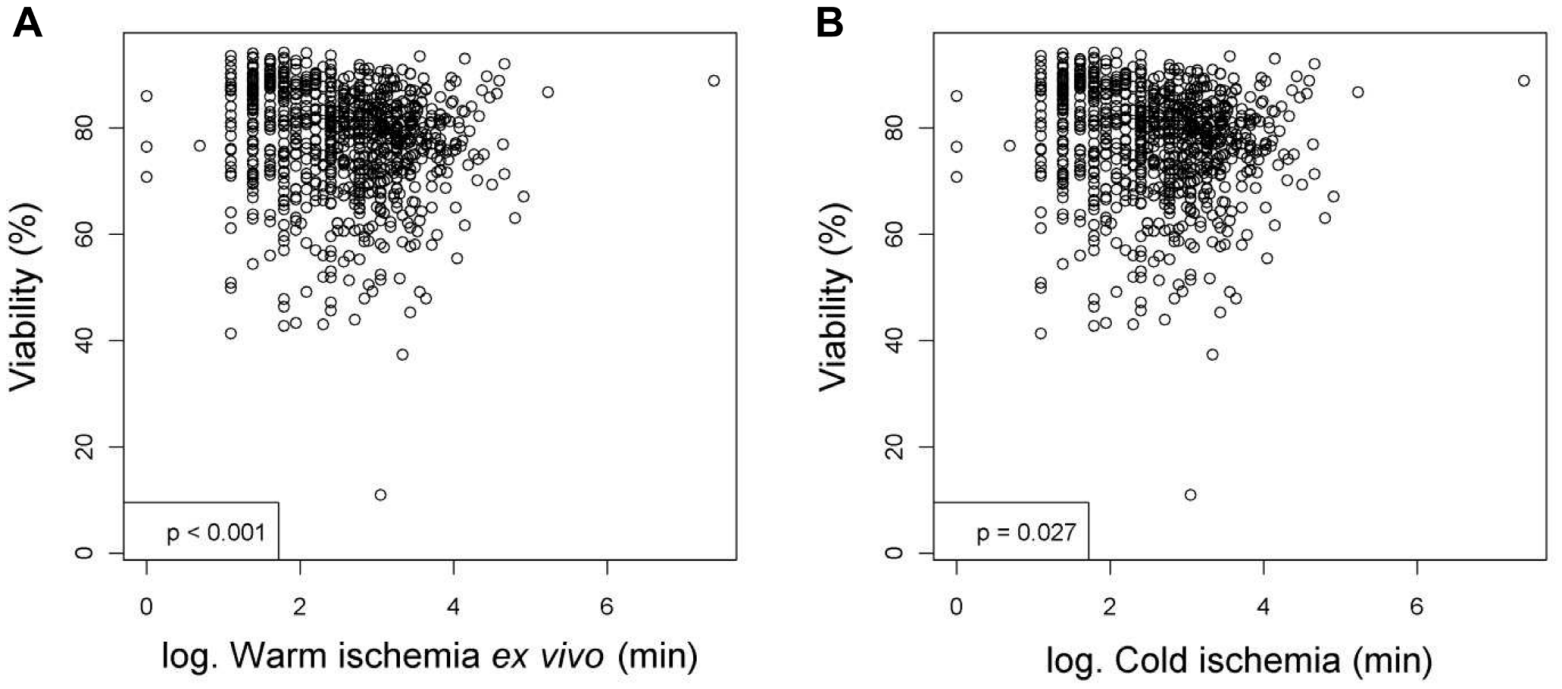
Tissue processing and cell isolation variables that have significant relationships with the viability (%) of hepatocytes after linear regression analyses. Figures show relationships between viability and (**A**) warm ischemia *ex vivo* (min) or (**B**) cold ischemia (min). Values were deemed significant when P<0.05.

**Figure 5:**
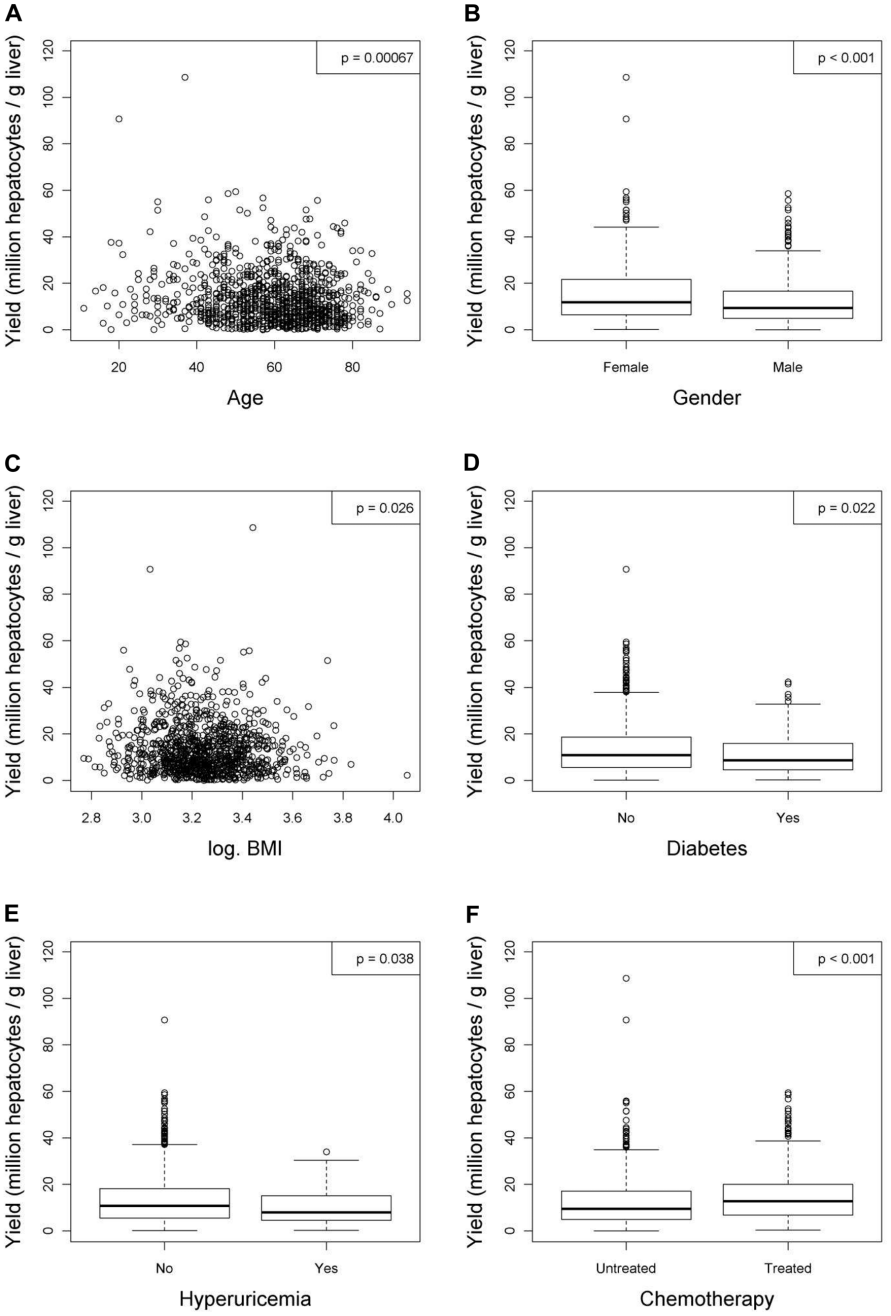
Donor characteristics that have significant relationships with the yield (million hepatocytes/gram liver) after linear regression analyses. Figures show relationships between yield and (**A**) age, (**B**) gender, (**C**) body mass index (BMI), (**D**) diabetes, (**E**) hyperuricemia or (**F**) chemotherapy. Values were deemed significant when P<0.05.

**Figure 6:**
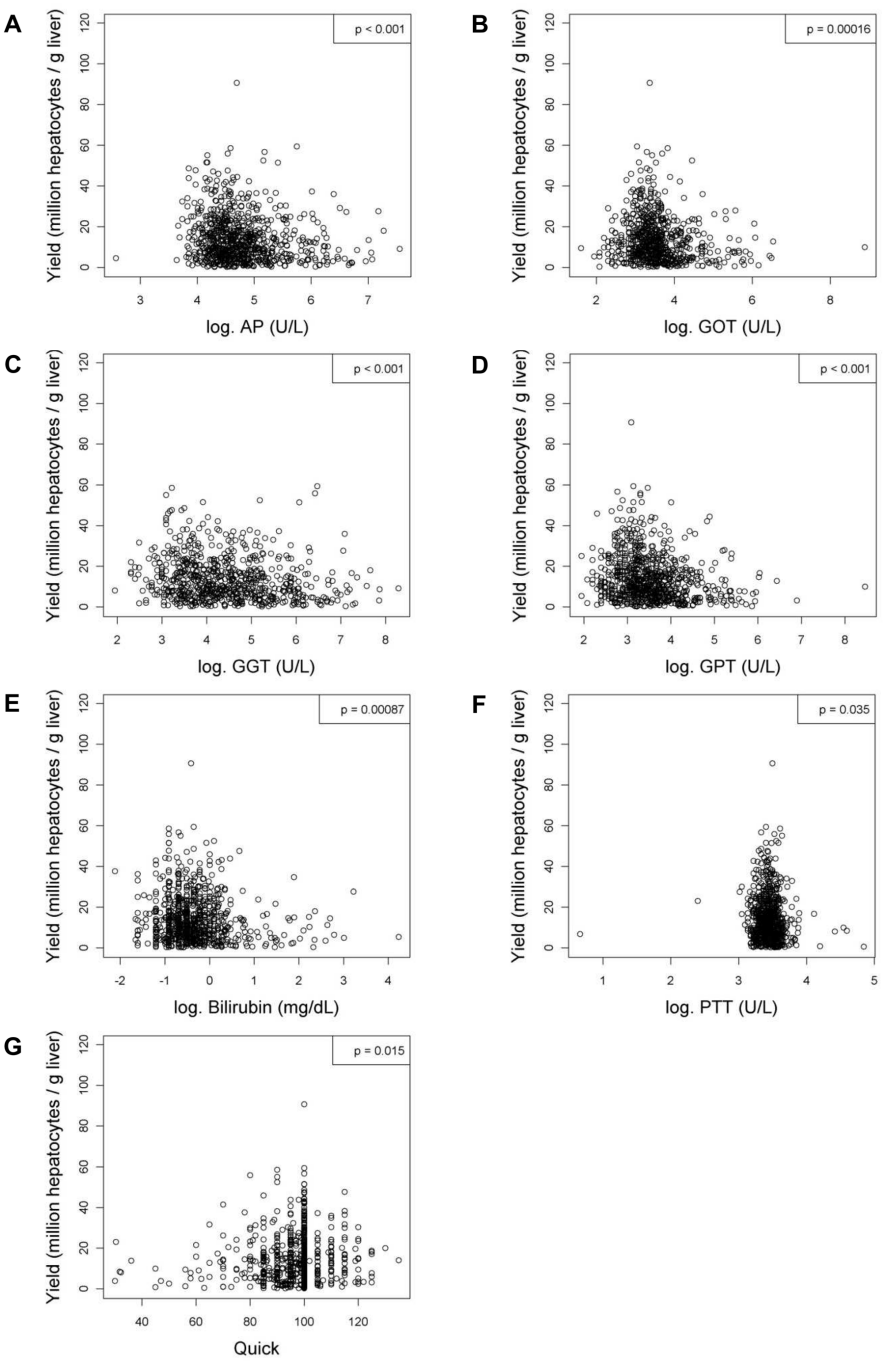
Variables measured in the blood or serum that have significant relationships with the yield (million hepatocytes/gram liver) after linear regression analyses. Figures show relationships between yield and (**A**) alkaline phosphatase activity (AP; U/L), (**B**) aspartate aminotransferase activity (GOT; U/L), (**C**) gamma-glutamyltranspeptidase activity (GGT; U/L), (**D**) alanine aminotransferase activity (GPT; U/L), (**E**) bilirubin (mg/dL), (**F**) partial thromboplastin time (PTT; s) or (**G**) quick value (%). Values were deemed significant when P<0.05.

**Figure 7:**
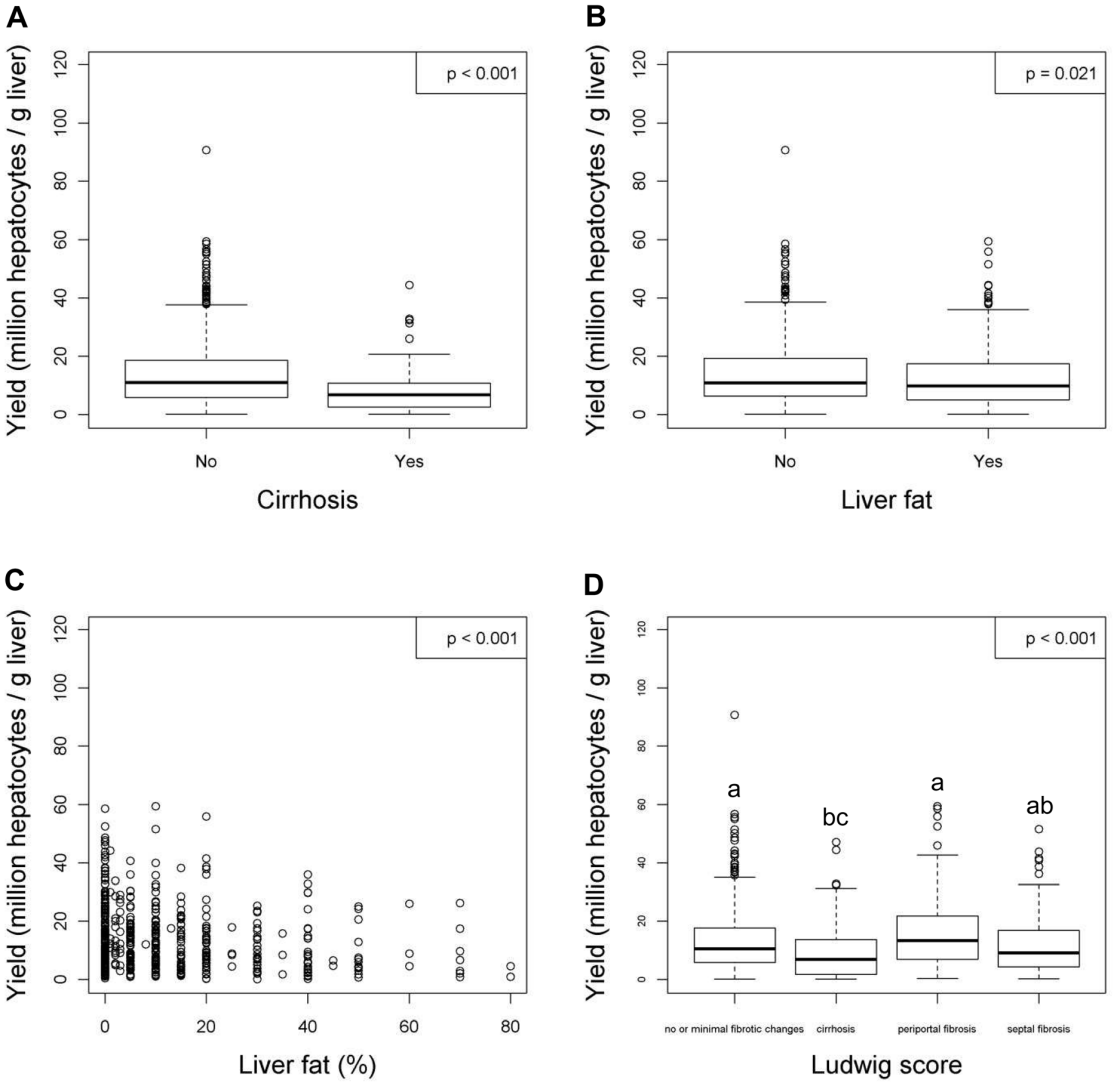
Liver variables that have significant relationships with the yield (million hepatocytes/gram liver) of hepatocytes after linear regression analyses. Figures show relationships between yield and (**A**) cirrhosis, (**B**) liver fat, (**C**) liver fat (%) or (**D**) Ludwig score. Values were deemed significant when P<0.05. For the variables of Ludwig score, operation type and surgical indication, variables not sharing the same alphabet are significantly different, *P*<0.05.

**Figure 8:**
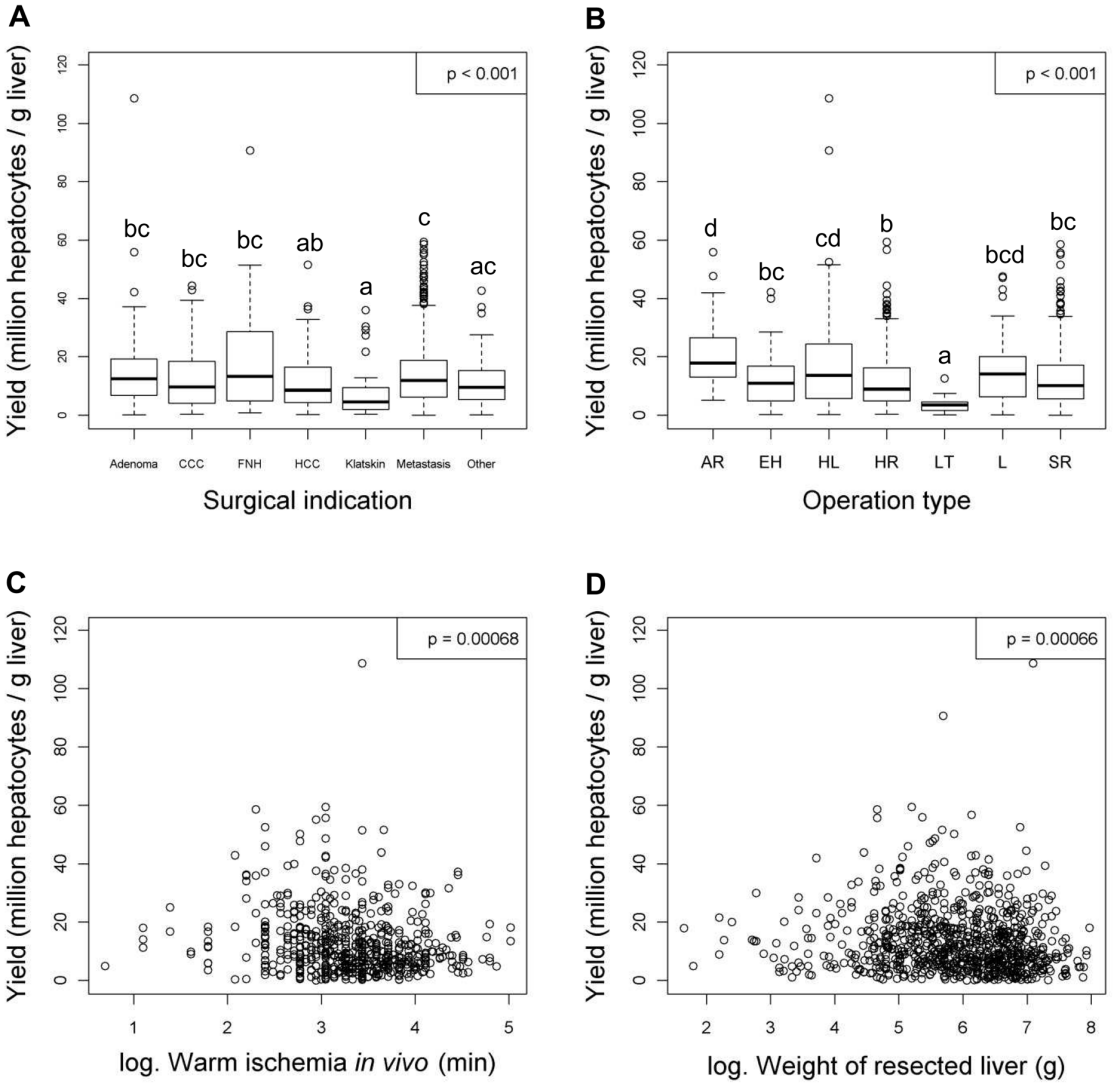
Operation variables that have significant relationships with the yield (million hepatocytes/gram liver) of hepatocytes after linear regression analyses. Figures show relationships between yield and (**A**) surgical indication, (**B**) operation type, (**C**) warm ischemia *in vivo* (min) or (**D**) weight of resected liver (g). Values were deemed significant when P<0.05. Abbreviations; hepatocarcinoma (HCC), focal nodular hyperplasia (FNH), cholangiocarcinoma (CCC), hemihepatectomy right (HR), hemihepatectomy left (HL), segment resection (SR), atypical resection (AR), extended hepatectomy (EH), lobectomy (L) and liver transplantation (LT).

**Figure 9:**
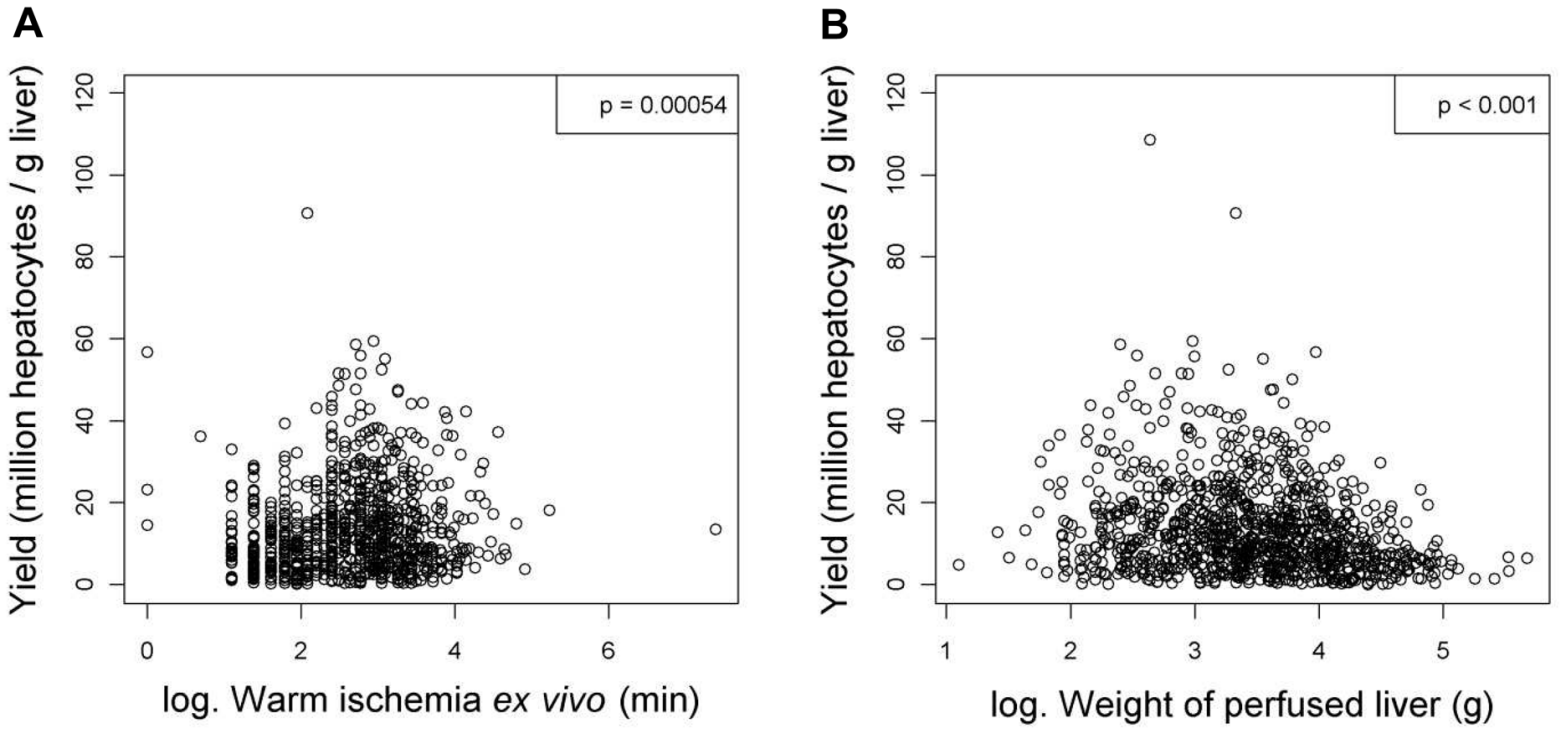
Tissue processing and cell isolation variables that have significant relationships with the yield (million hepatocytes/gram liver) of hepatocytes after linear regression analyses. Figures show relationships between yield and (**A**) warm ischemia *ex vivo* (min) or (**B**) weight of perfused liver (g). Values were deemed significant when P<0.05.

**Table 4:** The number of replicates (*N*) and the *P* values obtained after linear regression of the individual variables listed below to viability (%) or yield (million hepatocytes/g liver) of isolated human hepatocytes.

Variables	Viability^1^		Yield^2^	
	*N*	*P* value	*N*	*P* value
**Donor characteristics**				
Age	1030	0.027*	1026	0.00067*
Gender	1032	6.3×10^−7^ *	1028	1.6×10^−6^ *
Body mass index^3^	1006	0.022*	1002	0.026*
Fibrosis	910	0.0016*	905	0.074
Cirrhosis	907	0.76	902	1.1×10^−5^ *
Diabetes	1014	0.23	1009	0.022*
Obesity	1015	0.89	1010	0.44
Hypertension	1014	0.21	1009	0.052
Hypercholesterolemia	1012	0.32	1007	0.87
Hyperuricemia	1010	0.48	1005	0.037*
Smoking	573	0.93	569	0.091
Liver fat	886	0.00052*	881	0.021*
Liver fat (%)	522	0.47	517	3.6×10^−8^ *
Tumour type	995	0.51	991	0.063
Surgical indication	1017	0.053	1013	1.6×10^−6^ *
Chemotherapy	1027	0.31	1023	3.5×10^−5^ *
ASA physical status classification system	990	0.90	986	0.054
Ludwig score	813	0.0010*	809	7.5×10^−5^ *
**Clinical chemistry results before operation**				
Alkaline phosphatase activity (U/L)^3^	797	0.067	792	7.3×10^−5^ *
Aspartate aminotransferase activity (U/L)^3^	712	0.00012*	709	0.00016*
Gamma-glutamyltranspeptidase activity (U/L)^3^	690	0.0015*	687	4.2×10^−7^ *
Alanine aminotransferase activity (U/L)^3^	812	2.9×10^−5^ *	808	2.7×10^−9^ *
Cholinesterase activity (U/L)^3^	714	0.97	713	0.74
Bilirubin (mg/dL)^3^	810	0.0022*	805	0.00087*
Partial thromboplastin time (s)^3^	799	0.88	794	0.035*
Quick value (%)	803	0.027*	798	0.015*
**Operation parameters**				
Operation type	992	0.16	989	3.3×10^−8^ *
Warm ischemia *in vivo* (min)*^3^*	602	0.47	602	0.00068*
Warm ischemia *ex vivo* (min)^3^	888	3.9×10^−5^ *	887	0.00054*
Weight of resected liver (g)^3^	839	0.092	836	0.00066*
**Tissue processing and cell isolation parameters**				
Cold ischemia (min)^3^	913	0.027*	914	0.19
Weight of perfused liver (g)^3^	1030	0.68	1027	1.2×10^−11^ *

*Significant at *P*<0.05. Data are transformed to follow a normal distribution by logit^1^, fourth root^2^ or natural logarithm^3^ transformation.

**Table 5:** The regression coefficients (*β*), *P* values and *R^2^* numbers of variables after multivariate analyses for the dependent variable of viability (%) of isolated human hepatocytes.

Variables	Viability^1^	
	*β*	*P* value
**Donor characteristics**		
Fibrosis	−0.18	0.040*
Liver fat	−0.22	0.0065*
**Clinical chemistry results before operation**		
Gamma-glutamyltranspeptidase activity (U/L)^2^	−0.088	0.017*
Bilirubin	−0.17	0.0095*
***R^2^*** ** = 0.12, Intercept = 1.81**		

Variables presented are chosen by backward elimination.

*Significant at *P*<0.05, with *N* = 218. Data are transformed to follow a normal distribution by logit^1^ or natural logarithm^2^ transformation.

It was found that the viability of hepatocytes was decreased by increases in age, body mass index (BMI), aspartate aminotransferase (GOT) activity, gamma-glutamyltranspeptidase (GGT) activity, alanine aminotransferase (GPT) activity, bilirubin content in the blood, quick value, warm ischemia time *in vivo* and cold ischemia time (Table S1). In addition, the viability of hepatocytes was also decreased for males and for donors with fibrosis, liver fat or Ludwig scores indicating periportal fibrosis or septal fibrosis (Table S1).

In the case of the yield of hepatocytes, it was found that the yield was decreased by increases in age, BMI, liver fat, alkaline phosphatase (AP) activity, GOT activity, GGT activity, GPT activity, bilirubin content in the blood, partial thromboplastin time (PTT), warm ischemia time *in vivo* and weight of resected or perfused liver (Table S2). Further, the yield of hepatocytes was also decreased for males and for donors with cirrhosis, diabetes, hyperuricemia or certain surgical indications, operation types or Ludwig scores (Table S2). However, the yield of hepatocytes can be increased by increases in warm ischemia time *ex vivo*, Quick value and in donors treated with chemotherapy (Table S2).

### Multivariate analyses to determine the variables that affect the viability and the yield of hepatocytes

After multivariate analysis, the number of variables that have a significant effect on the viability of hepatocytes was reduced to 4 variables. It was found that the viability of hepatocytes was significantly decreased by the presence of fibrosis, liver fat and with increasing GGT activity and bilirubin content (Table 5).

For the yield of hepatocytes, it was found that yield was significantly decreased by the presence of liver fat and a Ludwig score indicating septal fibrosis. In addition, the yield of hepatocytes was decreased by increasing GOT activity, cold ischemia time and weight of perfused liver. However, the yield of hepatocytes was increased with chemotherapy treatment (Table 6).

**Table 6:** The regression coefficients (*β*), *P* values and *R^2^* numbers of variables after multivariate analyses for the dependent variable of yield (million hepatocytes/g liver) of isolated human hepatocytes.

Variables	Yield^1^	
	*β*	*P* value
**Donor characteristics**		
Liver fat (%)	−0.0069	0.0056*
Chemotherapy	0.19	0.0036*
Ludwig score		
-No or minimal fibrotic changes (reference)	-	-
-Periportal fibrosis	0.042	0.56
-Septal fibrosis	−0.21	0.047*
-Cirrhosis	−0.11	0.52
**Clinical chemistry results before operation**		
Aspartate aminotransferase activity (U/L)^2^	−0.11	0.016*
**Tissue processing and cell isolation parameters**		
Cold ischemia (min)^2^	−0.099	0.042*
Weight of perfused liver (g)	−0.16	0.0018*
**R^2^ = 0.32, Intercept = 3.14**		

Variables presented are chosen by backward elimination.

*Significant at *P*<0.05, with *N* = 128. Data are transformed to follow a normal distribution by fourth root^1^ or natural logarithm^2^ transformation.

### Generation of a model that allows for the calculation of projected viability and yield of hepatocytes

The information obtained from the multivariate analyses allowed the generation of formulae for the calculation of projected viability and the yield of isolated human hepatocytes as shown below.

### For the calculation of projected viability (%) of hepatocytes

**Formula 1.** Linear predictor of viability

 = *β*_0_+*β*_1_×log*_e_*(bilirubin)+*β*_2_×fibrosis+*β*_3_×liver fat+*β*_4_×log*_e_*(GGT+1)

The values of the various constants are as follows; *β*_0_ = 1.809, *β*_1_ = −0.169, *β*_2_ = −0.178, *β*_3_ = −0.216 and *β*_4_ = −0.088. While the continuous variables can be directly substituted with the absolute values recorded for a patient, categorical variables have to be substituted with “0” or “1”. For “fibrosis” – “0” for donors with no fibrosis and “1” for donors with fibrosis; for “liver fat” – “0” for donors with no liver fat and “1” for donors with liver fat.

**Formula 2.** Viability (%) = *e* ^(linear predictor of viability)^÷(1+*e* ^(linear predictor of viability)^)×100

### For the calculation of projected yield (million hepatocytes/g liver)

**Formula 3.** Linear predictor of yield

 = *β*_0_+*β*_1_×Chemotherapy+*β*_2_×log*_e_*(Cold ischemia+1)+*β*_3_×log*_e_*(Weight of perfused liver+1)+*β*_4_×Liver fat (%)+*β*_5_×(Ludwig score) Cirrhosis+*β*_6_×(Ludwig score) Periportal fibrosis+*β*_7_×(Ludwig score) Septal fibrosis+*β*_8_×log*_e_*(GOT activity+1)

The values of the various constants are as follows; *β*_0_ = 3.137, *β*_1_ = 0.19, *β*_2_ = −0.099, *β*_3_ = −0.159, *β*_4_ = −0.007, *β*_5_ = −0.108, *β*_6_ = 0.042, *β*_7_ = −0.211 and *β*_8_ = −0.114. While the continuous variables can be directly substituted with the absolute values recorded for a patient, categorical variables have to be substituted with “0” or “1”. For “chemotherapy” – “0” for donors with no chemotherapy and “1” for donors treated with chemotherapy; for the variable “Ludwig score”, the Ludwig score category of the a donor should be set to “1” while all other not applicable Ludwig score categories should be set to “0” for calculation. For example, if the donor has “cirrhosis”, this variable should be set to 1 at the same time as setting the variables “periportal fibrosis” and “septal fibrosis” to 0.

**Formula 4.** Yield (million hepatocytes/g liver) = linear predictor of yield^4^

### Validation of the models for calculating projected viability and yield of isolated hepatocytes

**Figure 10:**
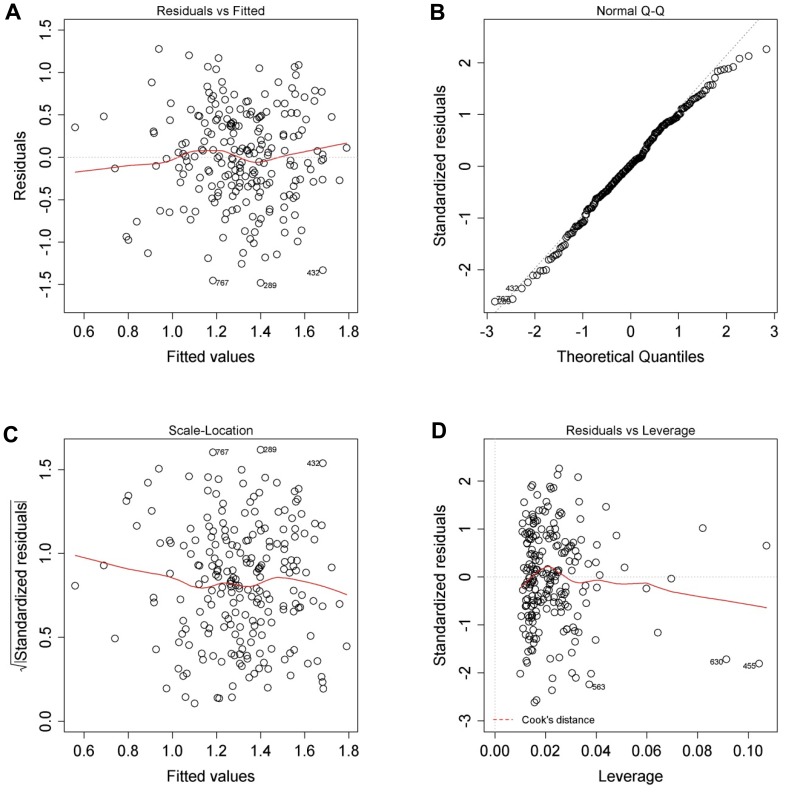
The model for calculating projected viability is appropriate. (**A**) Residuals versus fitted plot. (**B**) Normal quantile plot. (**C**) Square root of the standardised residuals versus fitted plot. (**D**) Standardised residuals versus leverage plot.

**Figure 11:**
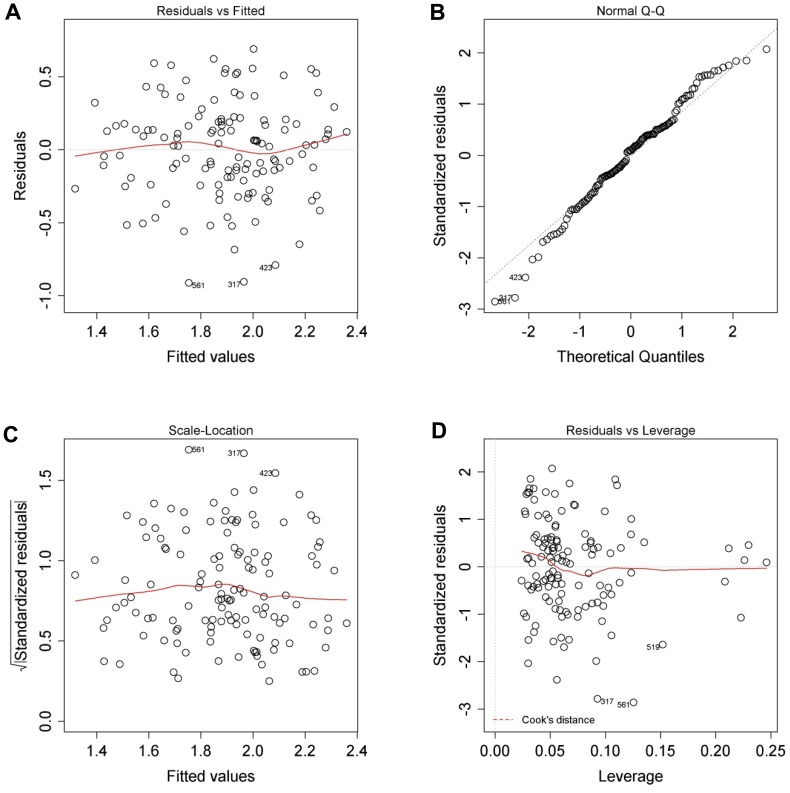
The model for calculating projected yield is appropriate. (**A**) Residuals versus fitted plot. (**B**) Normal quantile plot. (**C**) Square root of the standardised residuals versus fitted plot. (**D**) Standardised residuals versus leverage plot.

The appropriateness of the models for calculating the projected viability and yield of isolated hepatocytes are very similar and can be seen in Figures 10 and 11 respectively. Firstly, the residuals versus fitted plots (Figures 10A and 11A) show that there is no systematic relationship between the residuals and the predicted (or so-called fitted) values. These two figure panels (Figures 10A and 11A) also show that there are no systematic tendencies in the errors, such as heteroscedasticity etc.

Secondly, the normal quintile plots (Figures 10B and 11B) show that the residuals are approximately normally distributed. This distribution is necessary in order to obtain valid test statistics and *P* values of the regression coefficient.

Thirdly, the square root of the standardised residuals versus fitted plots (Figures 10C and 11C) show that there is no systematic relationship between the residuals and the predicted (or fitted) values. As before, these graphs also do not show systematic tendencies in the errors such as heteroscedasticity. However, in contrast to the residuals versus fitted plot, the standardised residuals were normalised once again, so that the residuals to have unit variance, using an overall measure of the error variance.

Finally, the standardised residuals versus leverage plots (Figures 10D and 11D) show the influence of regression results when leaving out a single observation from the dataset. Leverage can be used to detect multivariate outliers in the data, but in this case, the leverage is so small that no limits indicating big leverage of a single or several observations appear in our data.

## Discussion

This study aimed to determine donor characteristics, medical histories and operation, tissue processing and cell isolation parameters that affect the viability and the yield of isolated human hepatocytes. In order to do this, univariate analyses were first run to determine the variables that had a significant relationship to the viability or the yield of isolated human hepatocytes. Next, multiple regression analyses were run to determine the relative contributions of the various variables on the outcomes of viability and yield. From the results of the multiple regression analyses, a model was built to predict the viability and the yield of isolated hepatocytes (see Formulae 1–4 in results). Residual analyses were then done to check the regression assumptions in order to ensure that the model was appropriate.

This study has to the authors' knowledge, the largest number of donors examined for univariate analyses with 1034 donors and a sample size between 517 and 1032 for the individual variables. In contrast, other studies that carried out univariate analyses had sample sizes between 10 and 149 [9]–[15]. As a result, this study detects statistical significance in more variables than in the other studies (Tables 4, S1 and S2), probably due to an increase in statistical power. However, when multiple regression analyses were done, a reduced number of variables were found to be statistically significant. Typically, this is the case when multiple regression analyses are carried out, but it is important to consider that there is a loss of statistical power as the sample sizes were 218 for viability and 128 for yield of isolated hepatocytes as only cases with completed data on all the variables of interest were considered. However, as the other study that has conducted multiple regression analyses to the authors' knowledge has a sample size of 90 [16], this study still contributes useful information that will be discussed below.

When the absolute viability and yield numbers are considered, the hepatocytes isolated according to the authors' protocol [7] have a good balance of high viability (77±0.3) and yield (13.4±0.4) comparable to other groups with good results (Table 7). The comparison of variables found to have significant effects on the viability and yield of hepatocytes to what is known in the literature is challenging, as the results obtained by other groups are often contradictory (Tables 1 and 2). As such, the following paragraphs will instead focus on discussing the results of the multiple regression analyses.

**Table 7:** Viabilities (%) and yield (million hepatocytes per gram liver) of isolated human hepatocytes obtained by various groups.

Viability				Yield		*N*	Reference
Mean	Median	*N*	Reference	Mean	Median		
91±2	-	14	[11]	-	125	30	[41]
-	89	90	[16]	18.7±1.7	-	50	[15]
83±1	-	67	[12]	13.4±0.4	10.5	1028	Authors' own
83±1	-	72	[13]	10.6±7.8	-	41	[42]
80±8	-	41	[42]	8.2±5.7	-	42	[42]
78±0.3	-	10	[9]	7.9±1.2	-	14	[11]
77±0.3	79	1032	Authors' own	7.7±1.8	-	58	[13]
77±9	-	42	[42]	7.1±1.0	-	10	[9]
70±2	-	50	[15]	-	6.0	90	[16]
64±3	74	58	[39]	5.8±0.8	-	72	[13]
-	71	47	[14]	5.2±0.5	-	67	[12]
60±4	-	58	[13]	-	4.6	47	[14]
-	56	20	[38]	4.0±0.7	-	149	[10]
-	25	30	[41]	2.6±0.5	1.5	58	[39]

Values were expressed as means ± standard error of the mean.

After multiple regression analyses, this investigation has found that the viability of isolated human hepatocytes was significantly decreased by the presence of fibrosis, liver fat and with increasing GGT activity and bilirubin content (Formula 1). In the case of the yield of isolated human hepatocytes, it was found that the yield was significantly decreased by the presence of liver fat, a Ludwig score indicating septal fibrosis and by increasing GOT activity, cold ischemia time and weight of perfused liver. Further, the yield of hepatocytes was increased with chemotherapy treatment (Formula 3).

Increased cold ischemia time has been found to decrease the yield of isolated hepatocytes. In this study, the liver piece used for hepatocyte isolation was not perfused to remove the blood in the tissue before transport on ice to the laboratory. As a result, the formation of blood clots can occur in the tissue in the cases with longer transportation times even though the clotting process is slowed by low temperatures [17]. This could then affect the perfusion of the liver during the hepatocyte isolation process and decrease the yield. Yield is also decreased when the weight of the perfused liver is increased. Alexandre *et al*. [10] showed that the percentage of undigested tissue left after the isolation process is significantly increased in larger pieces of liver above 101 g. Also, the isolation set-up used in this study can provide a maximum of 8 cannulae for perfusing the liver piece [7]. Although the rate of perfusion per cannula is kept at similar levels independent of the size of the liver [7], it may be that portions of a larger liver are not perfused due to additionally available open blood vessels not being cannulated.

Fibrosis develops in response to many types of chronic liver diseases [18]. In such diseases, apoptosis plays a critical role both in liver injury and the subsequent fibrosis [18]. During the process of apoptosis, hepatocytes form apoptotic bodies that are phagocytosed by hepatic stellate cells resulting in an up-regulation of Transforming Growth Factor β (TGF-β) and procollagen α1, leading to subsequent inflammation and fibrogenesis [19]–[21]. Since fibrosis is closely linked with inflammation and cell death, it is not surprising that hepatocytes isolated from fibrotic livers have a significantly lower viability. In particular, this study found that septal fibrosis resulted in a significantly decreased yield of viable hepatocytes. The process of septal fibrosis begins with the extension of the septa between central veins through interhepatocellular space and the space of Disse on the sides of the sinusoids [22]. As fibrosis proceeds, capillarization and then venularization occurs [22], [23]. During this process, the fenestrations in some hepatic sinusoids are lost and the development of basal laminae occur alongside the collagenization of the extravascular spaces of Disse [22], [24]. This capillarization and fibrosis has been postulated to impair the leakage of macromolecules by creating a new barrier between the sinusoids and the hepatocytes [24]. This assertion has been supported by an approach utilising MRI, which shows that the extravascular distribution of high molecular weight contrast agents (6 or 52 kDa) is limited in a model of sinusoidal fibrosis [24]. These observations could explain the decreased yield obtained in this study due to septal fibrosis as collagenase that is 110 kDa will have limited access to the extracellular matrix in the direct vicinity of hepatocytes. Together with an increased amount of collagen surrounding capillaries that have to be digested, it is likely that the release of hepatocytes into a cell suspension is impaired and hence the reduced yield.

The presence of liver fat results in a significant decrease in the viability of hepatocytes. Hepatic steatosis, which is commonly found in obese or heavy alcohol drinkers, has been postulated to be a “first hit” that increases sensitivity to a “second hit” that could then trigger a cascade leading to steatohepatitis [25]. Steatohepatitis is characterised by inflammation and liver cell damage and could therefore lead to lower hepatocyte viability. Further, steatotic hepatocytes have been found to have increased sensitivity to hypoxic injury i.e. lower viability, due to the attenuation of Hypoxia Inducible Factor 1α expression and protein accumulation [26]. The presence of liver fat also results in a significant decrease in the yield of hepatocytes. It has been found that fat accumulation in hepatocytes results in microvascular alterations [27]. Various studies have demonstrated that lipid accumulation results in enlargement of hepatocytes, which widen the parenchymal cell plates, narrow and distort the lumens of the sinusoids and hence reduce the intrasinusoidal volume [27]. As a result, the sinusoids have impaired tissue perfusion and become poor conduits for conducting the collagenase-containing buffer used in the cell isolation process and possibly leading to a lower yield of hepatocytes. In addition, during centrifugation steps in the isolation process, steatotic hepatocytes tend to form a pellet less effectively, making it more likely that hepatocytes are lost when the supernatant is aspirated off (Authors' observation).

Levels of bilirubin and activities of GGT and GOT in the serum are standard assay parameters in liver function tests. Elevated levels of bilirubin and GGT, known indicators of hepatocyte injury [28], [29], have been found here to lower viability of isolated hepatocytes. Increased activity of GOT significantly decreased the yield of hepatocytes. This could be because a high level of GOT has been found to be a diagnostic marker for a number of diseases that can affect microcirculation or have increased collagen deposition, such as advanced alcoholic disease [30] or non-alcoholic chronic liver diseases with significant fibrosis [31], [32],

Hewes *et al.* [14] found that previous chemotherapy does not affect the median yield of isolated human hepatocytes (4.6 million viable cells per gram of liver). In contrast, this study found a significantly increased yield of hepatocytes from donors pre-treated with chemotherapy with median yields of 17 compared to 10 million viable cells per gram of liver from donors without chemotherapy. It could be possible that the analysis done here was able to pick up a significance due to an increased statistical power, as this study has 128 replicates compared to 47 replicates done in Hewes *et al.'*s study [14]. An additional support for this reasoning is that the univariate analysis done with 1027 replicates also indicated a statistical difference (*P* = 3.5×10^−5^). It is possible that chemotherapy has reduced extracellular matrix proteins, such as collagen, allowing for a more complete digestion of the liver piece with collagenase, which resulted in an increased yield of isolated hepatocytes. This is supported by the study of Drózdz and Kucharz [33], which found that a cytostatic drug, azathioprine caused a decrease in total collagen content in the liver. Further, Sorefenib, a drug that is approved for the treatment of hepatocarcinoma has been found to act as an antifibrotic agent that reduced collagen deposition in fibrosis models such as bile duct ligation, thioacetamide or dimethylnitrosamine administration in rats or carbon tetrachloride administration in mice [34]–[36]. Doxorubicin, another commonly used chemotherapeutic drug, also reduces collagen content in bile duct ligated rats by strongly inhibiting hepatic stellate cell proliferation [37].

In conclusion, this study has determined the variables that affect the viability and yield of isolated human hepatocytes. Further, this study has generated algorithms (Formulae 1–4) for the prediction of the viability or yield. A publicly accessible webpage (http://www.klinikum.uni-muenchen.de/Chirurgische-Klinik-und-Poliklinik-Grosshadern/de/0700-forschung/ag-leberregeneration/core-facility/Qualitaetsrechner_Hepatozyten.html) where researchers can key in their variables for the automatic calculation of projected viability and yield using JavaScript, has been made available for ease of use. It is hoped that this resource will prove useful for the selection of suitable donors for hepatocyte isolation and also as a reference so that procedural problems can be spotted due to an unanticipated lower viability and yield obtained.

## Supporting Information

Table S1 **The number of replicates (** ***N*** **),** ***P*** **values, multiple** ***R^2^*** **values (** ***R^2^*** **), intercepts, regression coefficients (β) obtained after linear regression of the individual variables to viability (%) of isolated human hepatocytes.** *Significant relationship of the indicated variable to hepatocyte viability, *P*<0.05. For the variable of Ludwig score, variables not sharing the same superscript alphabet are significantly different, *P*<0.05. Viability values were transformed to follow a normal distribution by the logit^1^.
(DOC)

Table S2 **The number of replicates (** ***N*** **),** ***P*** **values, regression coefficients (β), intercepts and multiple** ***R^2^*** **values (** ***R^2^*** **) obtained after linear regression of the individual variables to the yield (million/g liver) of isolated human hepatocytes.** *Significant relationship of the indicated variable to hepatocyte yield, *P*<0.05. For the variables of Ludwig score, operation type and surgical indication, variables not sharing the same superscript alphabet are significantly different, *P*<0.05. Yield values were transformed to follow a normal distribution by the fourth root^1^.
(DOC)
