# Supplementary material for: An Algorithm that Predicts the Viability and the Yield of Human Hepatocytes Isolated from Remnant Liver Pieces Obtained from Liver Resections
Source: PLoS One. 2014 Oct 14;9(10):e107567. doi: 10.1371/journal.pone.0107567 (PMC4196847; doi:10.1371/journal.pone.0107567)
Supplement: Table S1 — The number of replicates ( N ), P values, multiple R2 values ( R2 ), intercepts, regression coefficients (β) obtained after linear regression of the individual variables to viability (%) of isolated human hepatocytes. *Significant relationship of the indicated variable to hepatocyte viability, P<0.05. For the variable of Ludwig score, variables not sharing the same superscript alphabet are significantly different, P<0.05. Viability values were transformed to follow a normal distribution by the logit1. (DOC) [file pone.0107567.s001.doc]

| Variables | Viability^1^ | |  |  |  |
| --- | --- | --- | --- | --- | --- |
|  | *N* | *P* value | *R^2^* | Intercept | β |
| **Donor characteristics** | | | | | |
| Age | 1030 | 0.027* | 0.0048 | 1.51 | -0.0032 |
| Gender | 1032 | 6.3 x 10^-7^* | 0.024 | 1.43 | Female (reference), male (-0.20) |
| Log*_e_*(Body mass index) | 1006 | 0.022* | 0.0052 | 2.20 | -0.27 |
| Fibrosis | 910 | 0.0016* | 0.011 | 1.37 | No (reference), yes (-0.15) |
| Cirrhosis | 907 | 0.76 | 0.00011 | 1.33 | No (reference), yes (0.025) |
| Diabetes | 1014 | 0.23 | 0.0014 | 1.34 | No (reference), yes (-0.069) |
| Obesity | 1015 | 0.89 | 1.8 x 10^-5^ | 1.33 | No (reference), yes (0.0062) |
| Hypertension | 1014 | 0.21 | 0.0016 | 1.35 | No (reference), yes (-0.052) |
| Hypercholesterolemia | 1012 | 0.32 | 0.00099 | 1.34 | No (reference), yes (-0.069) |
| Hyperuricemia | 1010 | 0.48 | 0.00049 | 1.34 | No (reference), yes (-0.061) |
| Smoking | 573 | 0.93 | 0.00025 | 1.23 | No (reference), yes (-0.0041), ex-smoker (0.036) |
| Liver fat | 886 | 0.00052* | 0.014 | 1.39 | No (reference), yes (-0.14) |
| Liver fat (%) | 522 | 0.47 | 0.0010 | 1.27 | -0.0012 |
| Tumour type | 995 | 0.51 | 0.00044 | 1.36 | Benign (reference), malignant (-0.050) |
| Surgical indication | 1017 | 0.053 | 0.012 | 1.41 | Adenoma (reference), cholangiocarcinoma (-0.13), focal nodular hyperplasia (0.086), hepatocarcinoma (-0.12), klatskin (-0.39), metastasis (-0.075), others (-0.082) |
| Chemotherapy | 1027 | 0.31 | 0.0010 | 1.31 | Untreated (reference), treated (0.042) |
| ASA physical status classification system | 990 | 0.90 | 0.00061 | 1.37 | 1 (reference), 2 (-0.048), 3 (-0.056), 6 (-0.066) |
| Ludwig score | 813 | 0.0010* | 0.020 | 1.41 | No fibrosis (reference) (^a^), cirrhosis (-0.16^ab^), periportal fibrosis (-0.14^b^), septal fibrosis (-0.23^b^) |
| **Clinical chemistry results before operation** | | | | | |
| Log*_e_*(Alkaline phosphatase + 1) | 797 | 0.067 | 0.0042 | 1.58 | -0.061 |
| Log*_e_*(Aspartate aminotransferase + 1) | 712 | 0.00012* | 0.021 | 1.72 | -0.12 |
| Log*_e_*(Gamma-glutamyltranspeptidase + 1) | 690 | 0.0015* | 0.015 | 1.55 | -0.064 |
| Log*_e_*(Alanine aminotransferase + 1) | 812 | 2.9 x 10^-5^* | 0.021 | 1.69 | -0.11 |
| Log*_e_*(Cholinesterase + 1) | 714 | 0.97 | 2.0 x 10^-6^ | 1.28 | -0.0025 |
| Log*_e_*(Bilirubin +1) | 810 | 0.0022* | 0.012 | 1.37 | -0.15 |
| Log*_e_*(Partial thromboplastin time + 1) | 799 | 0.88 | 2.7 x 10^-5^ | 1.35 | -0.016 |
| Quick value | 803 | 0.027* | 0.0061 | 1.67 | -0.0038 |
| **Operation parameters** | | | | | |
| Operation type | 992 | 0.16 | 0.0094 | 1.33 | Atypical resection (reference), extended hepatectomy (-0.15), hemihepatectomy left (0.023), hemihepatectomy right (-0.058), liver transplant (0.20), lobectomy (0.080), segment resection (0.040) |
| Log*_e_*(Warm ischemia *in vivo* + 1) | 602 | 0.47 | 0.00086 | 1.26 | 0.030 |
| Log*_e_*(Warm ischemia *ex vivo* + 1) | 888 | 3.9 x 10^-5^* | 0.019 | 1.59 | -0.098 |
| Log*_e_*(Size of resected liver + 1) | 839 | 0.092 | 0.0034 | 1.54 | -0.037 |
| **Tissue processing and cell isolation parameters** | | | | | |
| Log*_e_*(Cold ischemia + 1) | 913 | 0.027* | 0.0053 | 1.55 | -0.067 |
| Log*_e_*(Size of perfused liver + 1) | 1030 | 0.68 | 0.00017 | 1.28 | 0.011 |
