# Supplementary material for: An Algorithm that Predicts the Viability and the Yield of Human Hepatocytes Isolated from Remnant Liver Pieces Obtained from Liver Resections
Source: PLoS One. 2014 Oct 14;9(10):e107567. doi: 10.1371/journal.pone.0107567 (PMC4196847; doi:10.1371/journal.pone.0107567)
Supplement: Table S2 — The number of replicates ( N ), P values, regression coefficients (β), intercepts and multiple R2 values ( R2 ) obtained after linear regression of the individual variables to the yield (million/g liver) of isolated human hepatocytes. *Significant relationship of the indicated variable to hepatocyte yield, P<0.05. For the variables of Ludwig score, operation type and surgical indication, variables not sharing the same superscript alphabet are significantly different, P<0.05. Yield values were transformed to follow a normal distribution by the fourth root1. (DOC) [file pone.0107567.s002.doc]

| Variables | Yield^1^ | |  |  |  |
| --- | --- | --- | --- | --- | --- |
|  | *N* | *P* value | *R^2^* | Intercept | β |
| **Donor characteristics** | | | | | |
| Age | 1026 | 0.00067* | 0.011 | 1.98 | -0.0032 |
| Gender | 1028 | 1.6 x 10^-6^* | 0.022 | 1.86 | Female (reference), male (-0.12) |
| Log*_e_*(Body mass index) | 1002 | 0.026* | 0.0049 | 2.35 | -0.17 |
| Fibrosis | 905 | 0.074 | 0.0035 | 1.81 | No (reference), yes (-0.056) |
| Cirrhosis | 902 | 1.1 x 10^-5^* | 0.021 | 1.81 | No (reference), yes (-0.24) |
| Diabetes | 1009 | 0.022* | 0.0052 | 1.80 | No (reference), yes (-0.088) |
| Obesity | 1010 | 0.44 | 0.00059 | 1.80 | No (reference), yes (-0.024) |
| Hypertension | 1009 | 0.052 | 0.0037 | 1.81 | No (reference), yes (-0.053) |
| Hypercholesterolemia | 1007 | 0.87 | 2.8 x 10^-5^ | 1.79 | No (reference), yes (0.0079) |
| Hyperuricemia | 1005 | 0.037* | 0.0043 | 1.80 | No (reference), yes (-0.12) |
| Smoking | 569 | 0.091 | 0.0084 | 1.85 | No (reference), yes (-0.011), ex-smoker (-0.16) |
| Liver fat | 881 | 0.020* | 0.0061 | 1.83 | No (reference), yes (-0.063) |
| Liver fat (%) | 517 | 3.6 x 10^-8^* | 0.057 | 1.90 | -0.0060 |
| Tumour type | 991 | 0.063 | 0.0035 | 1.88 | Benign (reference), malignant (-0.091) |
| Surgical indication | 1013 | 1.6 x 10^-6^* | 0.036 | 1.87 | Adenoma (reference) (^bc^), cholangiocarcinoma (-0.12^bc^), focal nodular hyperplasia (0.049^bc^), hepatocarcinoma (-0.18^ab^), klatskin (-0.36^a^), metastasis (-0.045^c^), others (-0.14^ac^) |
| Chemotherapy | 1023 | 3.5 x 10^-5^* | 0.017 | 1.75 | Untreated (reference), treated (0.11) |
| ASA physical status classification system | 986 | 0.054 | 0.0078 | 1.76 | 1 (reference), 2 (0.049), 3 (0.00093), 6 (-0.40) |
| Ludwig score | 809 | 7.5 x 10^-5^* | 0.027 | 1.80 | No fibrosis (reference) (^a^), cirrhosis (-0.20^bc^), periportal fibrosis (0.063^a^), septal fibrosis (-0.059^ab^) |
| **Clinical chemistry results before operation** | | | | | |
| Log*_e_*(Alkaline phosphatase + 1) | 792 | 7.3 x 10^-5^* | 0.020 | 2.26 | -0.092 |
| Log*_e_*(Aspartate aminotransferase + 1) | 709 | 0.00016* | 0.020 | 2.09 | -0.079 |
| Log*_e_*(Gamma-glutamyltranspeptidase + 1) | 687 | 4.2 x 10^-7^* | 0.037 | 2.13 | -0.071 |
| Log*_e_*(Alanine aminotransferase + 1) | 808 | 2.7 x 10^-9^* | 0.043 | 2.22 | -0.11 |
| Log*_e_*(Cholinesterase + 1) | 713 | 0.74 | 0.00015 | 1.79 | 0.015 |
| Log*_e_*(Bilirubin + 1) | 805 | 0.00087* | 0.014 | 1.89 | -0.12 |
| Log*_e_*(Partial thromboplastin time + 1) | 794 | 0.035* | 0.0056 | 2.35 | -0.16 |
| Quick value | 798 | 0.015* | 0.0073 | 1.53 | 0.0029 |
| **Operation parameters** | | | | | |
| Operation type | 989 | 3.3 x 10^-8^* | 0.045 | 2.09 | Atypical resection (reference) (^d^), extended hepatectomy (-0.32^bc^), hemihepatectomy left (-0.22^cd^), hemihepatectomy right (-0.34^b^), liver transplant (-0.80^a^), lobectomy (-0.23^bcd^), segment resection (-0.31^bc^) |
| Log*_e_*(Warm ischemia *in vivo* +1) | 602 | 0.00068* | 0.019 | 2.06 | -0.089 |
| Log*_e_*(Warm ischemia *ex vivo* +1) | 887 | 0.00054* | 0.013 | 1.65 | 0.056 |
| Log*_e_*(Size of resected liver + 1) | 836 | 0.00066* | 0.014 | 2.06 | -0.047 |
| **Tissue processing and cell isolation parameters** | | | | | |
| Log*_e_*(Cold ischemia +1) | 914 | 0.19 | 0.0019 | 1.71 | 0.026 |
| Log*_e_*(Size of perfused liver + 1) | 1027 | 1.2 x 10^-11^* | 0.044 | 2.20 | -0.12 |
